# A direct Primitive Variable Recovery Scheme for hyperbolic conservative equations: The case of relativistic hydrodynamics

**DOI:** 10.1371/journal.pone.0195494

**Published:** 2018-04-16

**Authors:** A. Aguayo-Ortiz, S. Mendoza, D. Olvera

**Affiliations:** 1 Instituto de Astronomía, Universidad Nacional Autónoma de México, AP 70-264, Ciudad de México 04510, México; 2 School of Mathematics, University of Bristol, Bristol BS8 1TW, United Kingdom; Public Library of Science, UNITED KINGDOM

## Abstract

In this article we develop a Primitive Variable Recovery Scheme (PVRS) to solve any system of coupled differential conservative equations. This method obtains directly the primitive variables applying the chain rule to the time term of the conservative equations. With this, a traditional finite volume method for the flux is applied in order avoid violation of both, the entropy and “Rankine-Hugoniot” jump conditions. The time evolution is then computed using a forward finite difference scheme. This numerical technique evades the recovery of the primitive vector by solving an algebraic system of equations as it is often used and so, it generalises standard techniques to solve these kind of coupled systems. The article is presented bearing in mind special relativistic hydrodynamic numerical schemes with an added pedagogical view in the appendix section in order to easily comprehend the PVRS. We present the convergence of the method for standard shock-tube problems of special relativistic hydrodynamics and a graphical visualisation of the errors using the fluctuations of the numerical values with respect to exact analytic solutions. The PVRS circumvents the sometimes arduous computation that arises from standard numerical methods techniques, which obtain the desired primitive vector solution through an algebraic polynomial of the charges.

## Introduction

The use of numerical methods to solve differential equations has constituted a substantial amount of work since the conception of approximate solutions to a given set of equations. In the last few decades, digital computers have been a great help to heavily iterate complicated partial differential equations using extensive numerical, parallel and adaptive mesh techniques in personal computers and large clusters.

Physical laws are often written in a set of conservative differential equations, for which there are many well established convergent numerical techniques to obtain accurate solutions. In spite of this, there is an intermediate step that is often, depending on the nature of the problem, extremely cumbersome to deal with. This appears since the general solution to the problem is obtained as a set of vector charges ***q*** at every point or cell on a given domain of space at a particular time in the iteration. However, physical phenomena are described and measured by means of a set of vector primitive variables ***u***. Depending on the nature of the physical problem, the function ***u***(***q***) may not have an analytic form and so, at every point or cell of the integration space a cumbersome technique requires to be performed for each time step. No matter how fast this routine may be, it introduces an extra computational time that can heavily grow when the space-time resolution increases. In problems of special relativistic hydrodynamics this fact appears and, at each time step, a 10th degree algebraic polynomial has to be solved for a unique given value of each component of the vector ***u*** (see e.g., for an excellent account on this, [1]).

To make things even more complicated, for each particular physical problem it is necessary to have either an analytic solution ***u***(***q***) or a specific numerical technique to obtain it.

In this article we show how it is possible to construct a general numerical iteration method, using a combination of finite differences and finite volume integration techniques for the time and spatial evolutions respectively, to directly find the solutions ***u*** avoiding any middle cumbersome step such as the ones mentioned above. This technique is so general that requires no analytical knowledge whatsoever of ***u***(***q***). The method developed is general and valid to any set of coupled conservative equations. We also show how this method can be applied in the particular case of 1D special relativistic hydrodynamics (1DRHD). For this particular case, we construct convergence tests.

The article is organised as follows. In the Appendix, we briefly mention some (mostly used in relativistic hydrodynamics for shock capturing) of the traditional methods to solve a set of conservative equations. In Section 1 we construct our “*Primitive Variable Recovery Scheme* (PVRS)” which can directly obtain the primitive variables from quite a standard numerical procedure. Section 4 deals with different convergence relativistic Sod [2] shock-tube tests and error estimates are given using a standard L_1_-norm. Also, the errors are graphically interpreted using the fluctuations of the solution with respect to analytical known values is presented. Finally, in Section 5 we discuss and conclude our results.

## 1 Primitive Variable Recovery Scheme (PVRS)

In the appendix we discuss some of the standard techniques for discretising any set of scalar and coupled conservative equations. This is done in order to easy understand the further developments of the article for the less expert reader, and not to interrupt the experienced one with such well known methods. However, we note that in the appendix and in what follows Einstein’s summation convention will be used throughout the equations displayed in this article, something that does not usually appear in the literature.

The usual way to solve a system of hyperbolic equations (cf. Eq (15)):

$$\begin{array}{c} \frac{\partial q}{\partial t}+\frac{\partial f(q)}{\partial x}=0, \end{array}$$ (1)

is by implementing Finite Difference and Finite Volume Methods (FDM & FVM) in order to obtain solutions for the conservative charges ***q***. In the particular case of relativistic and non-relativistic hydrodynamics, these charges are the linear momentum along the three dimensions *S*^*i*^, the energy *τ* and the particle density *D*. In order to compare the numerical solution with experiments and/or observations, a set of primitive physical measurable variables *u* needs to be constructed. For this particular case, this primitive variable set is given by by the pressure *p*, the velocity along three spacial dimensions *v*^*i*^ and, the particle number density *n*. Some authors prefer to find the particle mass density *ρ* rather than the particle number density *n*. For most practical proposes, both variables are related by *ρ* = *mn* where *m* is the average mass per particle. In here and in what follows all thermodynamical quantities (pressure *p*, particle number density *n* and energy density *e* and so on, are measured on its proper reference frame following the convention in [3, 4]). The explicit dependences ***q*** = ***q***(***u***(*x*, *t*)) and ***f*** = ***f***(***u***(*x*, *t*)) for 1D flow in the special relativistic case are given by (see e.g. [3, 4]):

$$\begin{array}{c} q_{1}=D=\frac{n}{\sqrt{1-v^{2}}}\;\text{and}\;f_{1}=v\frac{n}{\sqrt{1-v^{2}}}, \end{array}$$ (2)

$$\begin{array}{c} q_{2}=S^{x}=v\frac{e+p}{1-v^{2}}\;\text{and}\;f_{2}=v^{2}\frac{e+p}{1-v^{2}}+p, \end{array}$$ (3)

$$\begin{array}{c} q_{3}=\tau =\frac{e+v^{2}p}{1-v^{2}}\;\text{and}\;f_{3}=v\frac{e+p}{1-v^{2}}. \end{array}$$ (4)

where *e* is the total (rest plus internal) proper energy per unit volume which can be related with the density and pressure via a state equation *e* = *e*(*n*, *p*) like the one derived by Tooper [5] for a polytropic relativistic gas:

$$\begin{array}{c} e=nm+\frac{p}{\kappa -1}, \end{array}$$ (5)

where *κ* is the polytropic index. In the previous equations and in what follows we choose a system of units in which the velocity of light is set to unity.

As we can see from Eqs (2–4), obtaining the inverse function ***u*** = ***u***(***q***(*x*, *t*)) results in quite a completed algebraic problem. In fact, the solution to this problem leads to a system of transcendental algebraic equations that have been deeply studied by Riccardi [1]. One way of solving this system is by using a Newton-Raphson method (cf. [6]) but this or any other numerical solution to obtain ***u***(***q***(*x*, *t*)) will carry an extra error besides the proper numerical error of the FDM or FVM. This procedure also adds a bit of computational processing time since an iteration loop to find the solution needs to be carried out at each cell every time step. In order to avoid this cumbersome task, we show now how it is possible to obtain a direct numerical solution of the primitive variables, which is valid for all conservative equation systems (cf. Eq (15)).

## 2 PVRS attempts with finite difference methods

Let us begin by writing the system of *m* hyperbolic equations showing the explicit dependence on *m* primitive variables, i.e.:

$$\begin{array}{c} \frac{\partial q_{a}\left(u_{1},\ldots ,u_{m}\right)}{\partial t}+\frac{\partial f_{a}\left(u_{1},\ldots ,u_{m}\right)}{\partial x}=0. \end{array}$$ (6)

A necessary and sufficient condition for the existence of the solution *u*_1_, …, *u*_*m*_ is that *a* = 1, …, *m*. Now, using the chain rule, the above equation can be written in the following quasilinear form:

$$\begin{array}{c} \frac{\partial q_{a}}{\partial u_{b}}\frac{\partial u_{b}}{\partial t}+\frac{\partial f_{a}}{\partial u_{c}}\frac{\partial u_{c}}{\partial x}=0, \end{array}$$ (7)

where ∂***q***/∂***u*** and ∂***f***/∂***u*** are the Jacobian matrixes of the vectors ***q*** and ***f*** respectively. Multiplying the previous equation by the inverse matrix (∂***q***/∂***u***)^−1^ we get

$$\begin{array}{c} \frac{\partial u_{a}}{\partial t}+M_{ab}\frac{\partial u_{b}}{\partial x}=0, \end{array}$$ (8)

where

$$\begin{array}{c} M_{ab}≔{\left(\frac{\partial q_{c}}{\partial u_{a}}\right)}^{-1}(\frac{\partial f_{c}}{\partial u_{b}}). \end{array}$$ (9)

If we perform a discretisation of Eq (8) using a FDM (see e.g. Section Finite differences approach of the appendix), we obtain the following numerical expression:

$$\begin{array}{c} u_{a}\left(x_{i},t_{n+1}\right)=u_{a}\left(x_{i},t_{n}\right)-\frac{\Delta t}{2\Delta x}M_{ab}[u_{b}\left(x_{i+1},t_{n}\right)-u_{b}\left(x_{i-1},t_{n}\right)]. \end{array}$$ (10)

No matter how complicated the functional representations of ***q***(***u***) and ***f***(***u***), it is possible (if not by hand, using a Computer Algebra System) to compute the matrix *M*_*ab*_ only once before implementing a discretisation scheme. In what follows we show how to implement a numerical scheme to find directly the primitive variables ***u*** solving Eq (8). By doing this, the cumbersome step of recovering ***u*** from ***q*** at every cell for each time step is not needed anymore.

The discretisation Eq (10) is accurate to the first-order and yields quite good results on smooth solutions. When the solution contains a shock wave, the method is stable but not consistent and so no convergent. This could be understood because Eq (10) is mathematically similar to Eq (16) of the appendix [7] with the substitution of the vector ***u*** instead of ***q***. Furthermore, Eq (10) is written in a non-conservative form and so, the entropy and Rankine-Hugoniot jump conditions are not satisfied across the shock waves. Due to this fact, the obtained solution converges to a different weak solution as compared to the one obtained by a conservative method (see e.g. [8]). In other words, this FDM scheme does not work and the approach to follow is to consider flux contributions as in standard FVM.

## 3 Primitive Variable Recovery Scheme using combined FDM and FVM

We now show how to implement a Primitive Variable Recovery Scheme (PVRS) using both a FDM and a FVM schemes for the time and spatial evolution of the equations. As mentioned at the end of the previous section, the fluxes contribution in the method must not be altered because the entropy and Rankine-Hugoniot jump conditions must be accomplished. To do so, the spatial derivative term must be evolved using a Godunov-type method (e.g. an HLL-type Riemann solver).

In the appendix it is shown that the conservative set of Eq (1) can be discretised in the form of relation Eq (48), which can be written in a semi-discrete form as:

$$\begin{array}{c} \frac{\partial q_{a}\left(x_{i}\right)}{\partial t}=-\frac{1}{\Delta x}\left({\left[F_{a}^{HLL}\right]}_{i+1/2}^{n}-{\left[F_{a}^{HLL}\right]}_{i-1/2}^{n}\right), \end{array}$$ (11)

where ***F***^*HLL*^ stands for the HLL-type Riemann solver approximation for the spatial fluxes (see appendix). Using the chain rule on the left hand side of the previous equation, it follows that:

$$\begin{array}{c} \frac{\partial u_{a}\left(x_{i}\right)}{\partial t}=-\frac{A_{ab}\left(x_{i},t_{n}\right)}{\Delta x}({\left[F_{a}^{HLL}\right]}_{i+1/2}^{n}-{\left[F_{a}^{HLL}\right]}_{i-1/2}^{n}). \end{array}$$ (12)

where $A={\left(\partial q/\partial u\right)}^{-1}$. By applying a forward-difference formula scheme on the left hand side of Eq (12), we get

$$\begin{array}{c} u_{a}\left(x_{i},t_{n+1}\right)=u_{a}\left(x_{i},t_{n}\right)-\frac{\Delta t}{\Delta x}A_{ab}\left(x_{i},t_{n}\right)\left([F_{b}^{HLL}{]}_{i+1/2}^{n}-{\left[F_{b}^{HLL}\right]}_{i-1/2}^{n}\right). \end{array}$$ (13)

In Eq (13), we take a numerical flux approach as in standard FVM and a finite difference of the time derivative over the primitive variables ***u***. The approximate solution to the Riemann problem, where Rankine-Hugoniot’s condition take place, is the same as the one presented in the appendix HLL Riemann solver section. Furthermore, the characteristic velocities used in the HLL solver which correspond to the the eigenvalues of the Jacobian ∂***f***/∂***q***, can be computed either from matrix *M*_*ab*_ Eq (9) or from ∂*f*_*a*_/∂*q*_*b*_ since both matrixes are similar [7]. All matrixes and vectors $\left(M_{ab},A_{ab},f_{a},q_{a}\right)$ are computed using a piecewise reconstruction $\tilde{u}$ of the primitive variables, except for matrix *A*_*ab*_ which is evaluated on the midpoint *x*_*i*_ of the cell *C*_*i*_.

It is important to note that in Eqs (8) and (13) the second term on the right hand side has an implicit sum over the repeated index *a*.

Note that, although it seems that the PVRS discretisation Eq (13) arises directly from discretising the hybrid quasilinear equation ∂u/∂t+A∂f/∂x=0 –which can be directly obtained by using the chain rule on Eq (1), it is impossible to obtain the PVRS discretisation shown in Eq (13) using a standard conservative FVM as presented in the appendix, and which satisfies the entropy and Rankine-Hugoniot jump conditions.

By using Eq (13) on a numerical code, it would no longer be a concern to recover the primitive variables from the computed conservative charges; they would instead be solved directly! Therefore, it would not be necessary to create a module in the code to obtain the final required solution ***u***(*x*, *t*). In general terms, this procedure works out for any kind of conservative system in which ***q***(***u***(*x*, *t*)) and ***f***(***u***(*x*, *t*)) are at least given at some initial time.

The time step evolution of the Eq (13) that we use for our numerical simulations is given by the Method of Lines (MoL):

$$\begin{array}{c} \frac{\partial u(x_{i})}{\partial t}=-L\left(u\left(x_{i}\right)\right), \end{array}$$ (14)

where ***L***(***u***(*x*_*i*_)) is the right hand side of Eq (13) (see e.g. [9]), which can be further implemented with a Runge-Kutta integration.

## 4 Convergence test for PVRS in relativistic hydrodynamics

In this section we are going to show how this new method handles the evolution of a relativistic gas in a particular Riemann problem namely the *shock tube* (see e.g. [9]). This relativistic Sod [2] shock tube problem is a standard test that any code must fulfil for its validation. It has an exact analytical solution for both special relativistic and non-relativistic hydrodynamics and it is used for comparisons with numerical methods.

We calculated the numerical solution using PVRS discretisation Eq (13) with an approximate HLL Riemann solver, a *minmod* limiter for the reconstruction $\tilde{u}$ and a 4th order Runge-Kutta Method of Lines (MoL-RK4) for the integration. The problem was solved in the domain [0, 1] with *N* = 800 identical grid cells. We made three relativistic Sod tests with the initial discontinuity located at *x* = 0.5 and with initial states shown on Table 1. Furthermore, we compared the numerical results with the exact solution [9]. Also, we have estimated the usual L_1_-norm error for the following different resolutions: Δ*x*_1_ = 1/200, Δ*x*_2_ = 1/400, Δ*x*_3_ = 1/800, Δ*x*_4_ = 1/1600, Δ*x*_5_ = 3200 and Δ*x*_6_ = 1/6400.

**Table 1:** Initial parameters used for the relativistic Sod [2] shock tube tests described in the article. *κ* stands for the polytropic index.

Test	*p*_*L*_	*v*_*L*_	*n*_*L*_	*p*_*R*_	*v*_*R*_	*n*_*L*_	*κ*
1	1.0	0.0	1.0	0.1	0.0	0.125	4/3
2	13.33	0.0	10.00	0.1	0.0	1.0	4/3
3	1000	0.0	1.0	0.01	0.0	1.0	5/3

The time-step condition used in this method is different from the commonly used by many authors (cf. [10]). A general CFL-condition applied to this numerical scheme was constructed by us and used in the set of examples presented. The exact condition and its derivation is a subject beyond the scope of this article and will be published elsewhere. For practical purposes, the time step interval can be chosen as a sufficiently smaller number than the corresponding CFL condition (cf. Eq (40)). For the examples presented below, we have chosen a fixed time step for each simulation.

**4.0.1 Test 1**: **Weak relativistic blast wave**

**Fig 1:**
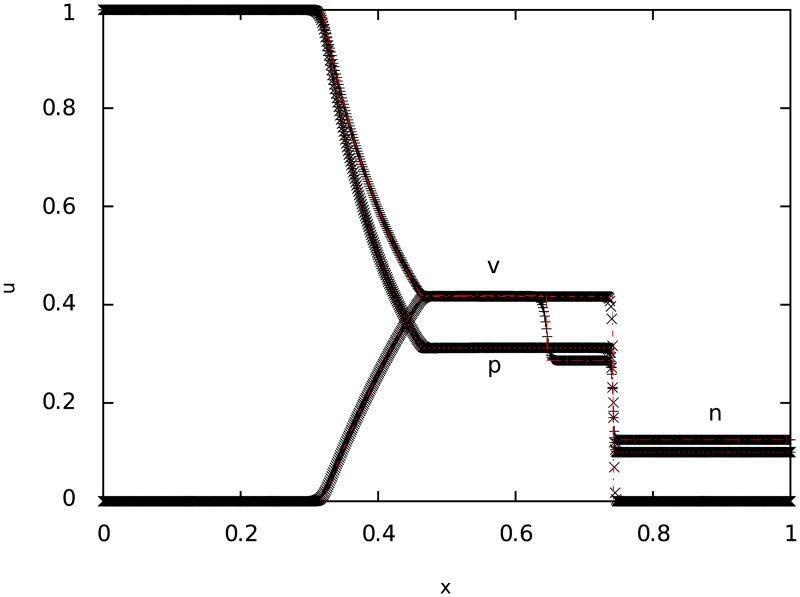
Test 1. The figure shows the result of the simulation of a weak relativistic (Sod shock tube) blast wave explosion at t = 0.35 for the particle number density *n*, pressure *p* and velocity *v*. The time step used for the simulation was 0.001.

The first test corresponds to a lowly relativistic blast wave explosion. The results can be seen in Fig 1, where we compare the numerical solution (points) with the exact solution (lines). It is clear that for both, smooth parts and discontinuities, the numerical solution converges quite well to the exact one.

**4.0.2 Test 2**: **Mildly relativistic blast wave**

**Fig 2:**
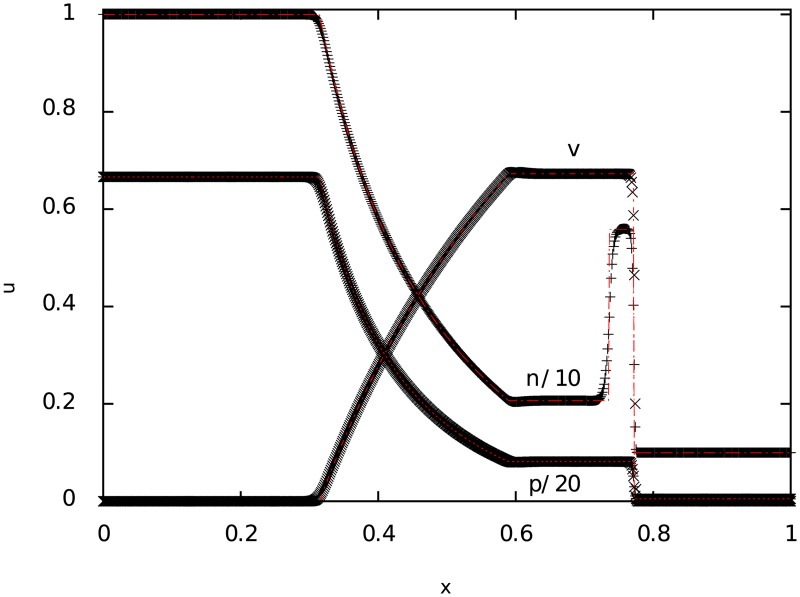
Test 2. The figure shows the result of a mildly relativistic (Sod shock tube) blast wave explosion at t = 0.35 for particle number density *n*, pressure *p* and velocity *v*. The time step used for the simulation was 0.001.

The second test corresponds to a mildly relativistic blast wave explosion. The results can be seen in Fig 2, where we compare the numerical solution (points) with the exact one (lines). The importance of this test is to see if, with a relative high difference in pressure between both states, the numerical method is capable of solving the density function at the contact discontinuity.

**4.0.3 Test 3**: **Strong relativistic blast wave**

**Fig 3:**
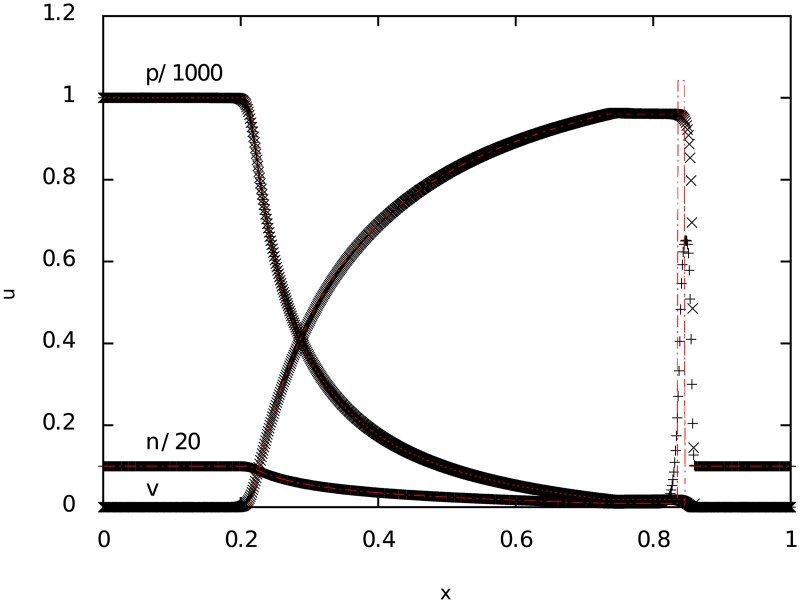
Test 3. The figure shows the result of a strong relativistic (Sod shock tube) blast wave explosion at t = 0.35 for particle number density *n*, pressure *p* and velocity *v*. The time step used for the simulation was 0.0001.

Finally, the last test corresponds to a strongly relativistic blast wave explosion. In this case, the density discontinuity is produced by a a 5 orders of magnitude difference between right and left initial detonation pressure, creating a thin shell which numerically is harder to resolve at low resolutions. However, with a relatively small number of cells and a weak variable reconstruction, the results shown on Fig 3 are as good as the ones obtained by other codes (cf. [10, 11]).

### 4.1 Error estimates

We have calculated the error of each test using the traditional L_1_—norm value. The convergence order of this test is given by log(*error*_*i*_/*error*_*i*−1_)/*log*(1/2), where *error*_*j*_ is the L_1_—norm of the Δ*x*_*j*_ resolution. As we can see from Table 2, the error decreases when the resolution increases, as expected. Also, we obtain first order convergence for all test in at least one resolution. Additionally, we made an experiment following [6] of a static Gaussian curve in order to estimate the order of convergence of a smooth static profile which, for this case, reaches a convergence value of about 2 in all the tested resolutions for a fixed time step of 0.01. As expected, this means that the important error of the relativistic Sod shock tube test relays on the discontinuities. This is the reason as to why we consider that taking the L_1_—norm is not a clear indicator of the “real” error at the shock waves, so we propose a more relevant useful visual interpretation of this estimation as follows.

**Table 2:** The L_1_-norm for the error in the numerical density for the *minmod* limiter with different numerical resolutions. The L_1_-norm is computed for all shock-tube and Gaussian tests at time *t* = 0.35. We also show the order of convergence between different resolutions. Since the error decreases when the resolution increases, the PVRS constructed in the article is stable and converges to the exact solution. The data of this is presented in S1 File.

	Error				Order of Convergence			
Resolution	Test 1	Test 2	Test 3	Smooth	Test 1	Test 2	Test 3	Smooth
Δ*x*_1_	3.83e-3	9.00e-2	1.93e-1	4.74e-4	-	-	-	-
Δ*x*_2_	2.12e-3	5.04e-2	1.60e-1	1.28e-4	0.85	0.84	0.27	1.89
Δ*x*_3_	1.21e-3	2.61e-2	1.21e-1	0.34e-4	0.81	0.95	0.40	1.91
Δ*x*_4_	6.68e-4	1.51e-2	8.03e-2	0.09e-4	0.85	0.79	0.59	1.92
Δ*x*_5_	4.01e-4	1.02e-2	4.56e-2	0.02e-4	0.74	0.57	0.81	2.17
Δ*x*_6_	2.22e-4	5.07e-3	2.62e-2	-	0.83	1.00	1.05	-

**Fig 4:**
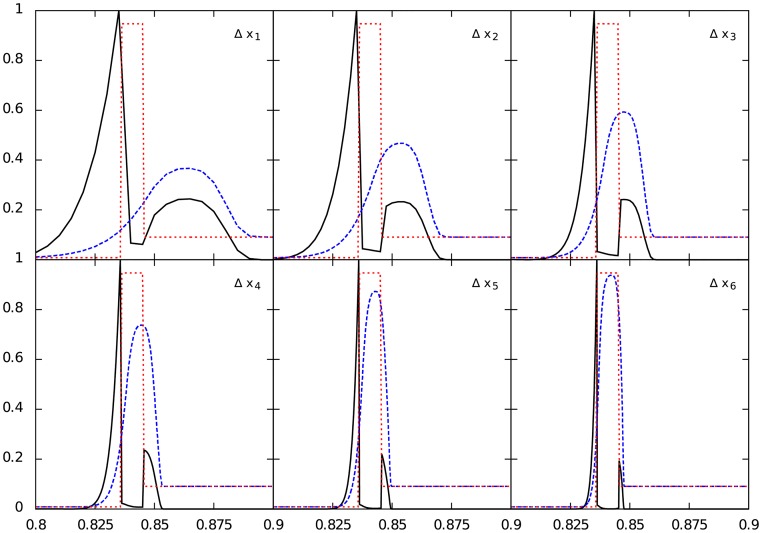
Comparison between the exact (red dashed-line) and the numerical solution (blue dashed-line) of the contact discontinuity in density for the Test 3 vs. the fluctuation |*u*_num_ − *u*_exact_|/*u*_exact_ (black continuous line) at each point, for all the tested resolutions. Note that as the resolution increases, the width of the fluctuation decreases, showing the convergence in a straightforward manner.

In Fig 4 we show both exact (red dashed-line) and numerical (blue dashed-line) solution vs. the fluctuation |*u*_num_ − *u*_exact_|/*u*_exact_ at each point (black line), for the density in Test 3 at every resolution. We can see how the Full Width at Half Maximum (FWHM) of the fluctuation tends to zero as the resolution increases. Working with the fluctuation of the numerical solution about the exact solution is a much better way to easily see the convergence of a numerical method, rather than the traditional L_1_-norm for which smoothing of the errors can be wrongly interpreted as a positive convergence test.

## 5 Discussion

In this article we have developed a new numerical algorithm to solve any set of coupled differential conservative equations for which the primitive variable vector ***u*** is directly obtained. This is a forward step in numerical methods, since it avoids any intermediate step reconstruction of the primitive variable vector from a previously obtained charge vector ***q*** at all points or cells in space at each time. In principle, this means that numerical codes can be written in a more direct form. Also, depending on the nature of the physical problem to solve, the computational time may be reduced with this technique.

For practical purposes, we always had in mind special relativistic hydrodynamical problems and for this reason the specific techniques used throughout the article deal with hydrodynamical shock capturing schemes. We demonstrated in the article that the Primitive Variable Recovery Scheme (PVRS) showed good convergence for three shock-tube and one Gaussian tests. Further explorations in other directions, such as a non-static Gaussian test [12] need to be investigated. We will explore more details in future works.

The PVRS presented in this article can be implemented straightforward to any standard hydrodynamical code that already uses HLL Riemann solvers given by Eq (13).

In summary, the PVRS is a numerical maneuver to circumvent the embroiling construction of the primitive vector once the charge vector is obtained from any standard procedure used to solve a set of coupled conservative equations in physical systems.

We are constructing a GNU Public Licensed (GPL) free software (http://www.gnu.org) called “*aztekas*” (http://www.aztekas.org) that deals with relativistic hydrodynamics using this PVRS technique.

## Appendix

### Traditional approach for numerically solving conservative equations

In this appendix, we deal with traditional well known methods for solving conservative equations. Our intention is to briefly introduce the less versed reader to this topics using Einstein’s summation convention.

A system of *m* conservative equations in one dimension is usually written as:

$$\begin{array}{c} \frac{\partial q_{a}}{\partial t}+\frac{\partial f_{a}\left(q_{1},\ldots ,q_{m}\right)}{\partial x}=0, \end{array}$$ (15)

where the subindex *a* takes values from 1 to *m*, ***q*** ≔ ***q***(***u***(*x*, *t*)) is the vector of *conservative charges* and ***f*** ≔ ***f***(***q***(***u***(*x*, *t*))) is the corresponding *flux* vector along the *x* axis at a given time *t*. The vector ***u*** corresponds to the *primitive variables* for which its number of entries and functional form of ***q***(***u***) depends on the particular problem to solve. From this point onwards, we are going to use ***f***(*x*, *t*) instead of the cumbersome notation ***f***(***q***(***u***(*x*, *t*))), bearing in mind that both, charges and flux vectors, depend on the primitive variables ***u***(*x*, *t*). As it is shown in section 1, the fluxes also have an explicit dependence on the primitive variables but are usually written in terms of the conservative charges.

We can rewrite Eq (15) in the *quasilinear* following form

$$\begin{array}{c} \frac{\partial q_{a}}{\partial t}+J_{ab}\frac{\partial q_{b}}{\partial x}=0, \end{array}$$ (16)

where *J*_*ab*_ is the *Jacobian* matrix of ***f***(***q***). From now on, we use Einstein implicit sum convention over two repeated subindexes contained in the set {*a*, *b*, *c*, *d*}. If the Jacobian matrix satisfies the conditions of having real eigenvalues and a set of independent eigenvectors, then we say that the system Eq (15) is *hyperbolic* (see e.g. [8]).

In the linear cases (when ***f*** is a linear function of ***q***), there exists an analytical solution for (15), but many physical cases give rise to nonlinear conservative systems which are required to be solved using numerical methods.

In the following subsections we briefly mention two of the main numerical methods used to solve 1D conservative systems such as the one written in Eq (15).

### Finite differences approach

The finite differences method (FDM) is one of the most useful and simple numerical methods for solving ordinary and partial differential equations. It consists of an approximation of the derivatives of fluxes and charges based on approximations of their values on sufficiently small intervals of space and time. The space is divided in a grid of *N* centred points spaced by equal length Δ*x* intervals in which the equation is evaluated.

Using Taylor expansions of the involved quantities, it is possible to work out the finite difference form of Eq (15) to find the value of ***q*** in all the grid at time *t* + Δ*t* ≕ *t*_*n*+1_ based on its value at *t* ≕ *t*_*n*_:

$$\begin{array}{c} q_{a}\left(x_{i},t_{n+1}\right)=q_{a}\left(x_{i},t_{n}\right)-\frac{\Delta t}{2\Delta x}[f_{a}\left(x_{i+1},t_{n}\right)-f_{a}\left(x_{i-1},t_{n}\right)], \end{array}$$ (17)

where *x*_*i*_ is the *i*-th point on the grid. This is the Forward Time Central Space (FTCS) Euler method [8]. In Eq (17), the derivative ∂*f*_*a*_/∂*x* at a given time *t*_*n*_ was written using a central approximation value given by (*f*_*a*_(*x*_*i*+1_) − *f*_*a*_(*x*_*i*−1_))/(2Δ*x*). For the left and right boundary points this derivative can be written using a right or left derivative approximation given by: (*f*_*a*_(*x*_1_) − *f*_*a*_(*x*_0_))/Δ*x* and (*f*_*a*_(*x*_*N*−1_) − *f*_*a*_(*x*_*N*_))/Δ*x* respectively. Unfortunately, Eq (17) leads to numerical unstable solutions [13]. To overcome this problem, many higher order methods have been developed and successfully implemented over time [14].

When a second-order finite differences approximation method is used, additional source *artificial viscosity* terms appear in Eq (17). Those additional terms are either due to the second derivative approximation in Taylor series or to second differences approximation of the first derivatives (see e.g. [14]). The artificial viscosity name was given by von Neumann [13] since it resembles the viscosity term of the Navier-Stokes equation, but has nothing to do with any physical viscosity.

The general form of the artificial viscosity can be written as [14]:

$$\begin{array}{c} \begin{array}{c} q_{a}\left(x_{i},t_{n+1}\right)=q_{a}\left(x_{i},t_{n}\right)\\ \;-\frac{\Delta t}{2\Delta x}\left[f_{a}\left(x_{i+1},t_{n}\right)-f_{a}\left(x_{i-1},t_{n}\right)\right]\\ \;+\frac{\Delta t}{2\Delta x}[\epsilon_{a}^{+}\Delta q_{a}^{+}\left(x_{i},t_{n}\right)-\epsilon_{a}^{-}\Delta q_{a}^{-}\left(x_{i},t_{n}\right)], \end{array} \end{array}$$ (18)

where $\epsilon_{a}^{\pm }$ are the *coefficients of second-order explicit artificial viscosity* and $\Delta q_{a}^{\pm }\left(x_{i},t_{n}\right)=\pm q\left(x_{i\pm 1},t_{n}\right)\mp q\left(x_{i},t_{n}\right)$. The choice $\epsilon_{a}^{\pm }=2\Delta x/\Delta t$ simplifies the above equation to:

$$\begin{array}{c} \begin{array}{c} q_{a}\left(x_{i},t_{n+1}\right)=\frac{1}{2}\left(q_{a}\left(x_{i+1},t_{n}\right)+q_{a}\left(x_{i-1},t_{n}\right)\right)\\ -\frac{\Delta t}{2\Delta x}[f_{a}\left(x_{i+1},t_{n}\right)-f_{a}\left(x_{i-1},t_{n}\right)], \end{array} \end{array}$$ (19)

which is known as the *Lax-Friedrich method*. Other second-order-two-step methods, such as the *Lax-Wendroff method*, have been developed and successfully implemented in many numerical codes.

**Fig 5:**
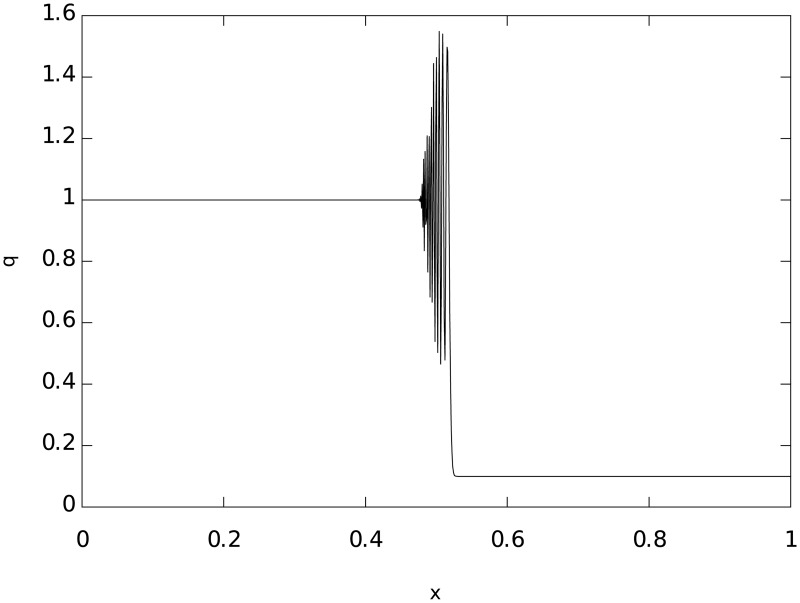
The graph shows the numerical solution of the advection equation: ∂_*t*_ *q* + ∂_*x*_ *q* = 0, using exclusively the MacCormack method. The solution shows non-physical oscillations in a finite-jump discontinuity due to the Gibbs phenomenon. At later times, the oscillations grow breaking even more the expected solution. The graph was constructed using the initial conditions of *q* = 1.0 if *x* < 0.5 and *q* = 0.125 elsewhere at a fixed time *t* = 0.03.

One such favourite two-step method was proposed by MacCormack [15]. It makes a *forward-prediction* of ***q*** and with it, a *backward-correction*:

$$\begin{array}{c} {\tilde{q}}_{a}\left(x_{i},t_{n}\right)≔q_{a}\left(x_{i},t_{n}\right)-\frac{\Delta t}{\Delta x}[f_{a}\left(x_{i+1},t_{n}\right)-f_{a}\left(x_{i},t_{n}\right)], \end{array}$$ (20)

$$\begin{array}{c} q_{a}\left(x_{i},t_{n+1}\right)=\frac{1}{2}\{q_{a}\left(x_{i},t_{n}\right)+{\tilde{q}}_{a}(x_{i},t_{n})-\frac{\Delta t}{\Delta x}\left[{\tilde{f}}_{a}\left(x_{i},t_{n}\right)-{\tilde{f}}_{a}\left(x_{i-1},t_{n}\right)\right]\}. \end{array}$$ (21)

where $\tilde{f}≔f\left(\tilde{q}\right)$. This method has been proved to be consistent, convergent and stable which is the requirement for any numerical method used in a computational code. Nevertheless, in discontinuities and regions with high pressure gradients, such as regions with shock-waves, this algorithm introduces a dispersive error called the Gibbs phenomenon, which consists on the presence of large spurious oscillations near the finite-jump, such as the example shown in Fig 5.

To solve this problem, it is common to apply a *corrective diffusion* in the regions where the non-physical oscillations appear. The correction presented by Book [16] is

$$\begin{array}{c} q_{a}^{*}\left(x_{i},t_{n}\right)=q_{a}\left(x_{i},t_{n}\right)+\eta \left[q_{a}\left(x_{i+1},t_{n}\right)-2q_{a}\left(x_{i},t_{n}\right)+q_{a}\left(x_{i-1},t_{n}\right)\right], \end{array}$$ (22)

where *η* is the *antidiffusion coefficient* at space-time points *x*_*i*_ and *t*_*n*_:

$$\begin{array}{c} \eta =\{\begin{array}{cc} \eta_{0}\leq 1/4, & \text{if}\;\left(\Delta q_{a}^{+}\right)\left(\Delta q_{a}^{-}\right)<0,\\ 0, & \text{if}\;\left(\Delta q_{a}^{+}\right)\left(\Delta q_{a}^{-}\right)>0. \end{array} \end{array}$$ (23)

### Finite volume approach

A more natural way of obtaining the discretisation form of (15) is the Finite Volume Method (FVM) which is based on a subdivision of the spatial domain into intervals (also called *control volumes* or *grid cells*) *C*_*i*_ ≔ [*x*_*i*−1/2_, *x*_*i*+1/2_]. The integration of Eq (15) over *C*_*i*_ between times *t*_*n*_ and *t*_*n*+1_ yields:

$$\begin{array}{c} \int_{t_{n}}^{t_{n+1}}\int_{C_{i}}[\frac{\partial q_{a}\left(x,t\right)}{\partial t}+\frac{\partial f_{a}\left(x,t\right)}{\partial x}]\text{d}x\;\text{d}t=0. \end{array}$$ (24)

The integral of ∂_*t*_ *q* over time and the integral of ∂_*x*_ *f* over space can be solved exactly and so, the next integral form of the previous equation is found:

$$\begin{array}{c} \begin{array}{cc} & \int_{C_{i}}\left(q_{a}\left(x,t_{n+1}\right)-q_{a}\left(x,t_{n}\right)\right)\text{d}x\\ + & \int_{t_{n}}^{t_{n+1}}\left(f_{a}\left(x_{i+1/2},t\right)-f_{a}\left(x_{i-1/2},t\right)\right)\text{d}t=0. \end{array} \end{array}$$ (25)

At this point, both integrals in the previous equation cannot be integrated unless we have the exact form of *q*, which is precisely the solution to the problem. In order to overcome this, we define each integration as a new numerical vector in the following form:

$$\begin{array}{c} {\left[Q_{a}\right]}_{i}^{n}=\frac{1}{\Delta x}\int_{C_{i}}q_{a}\left(x,t_{n}\right)\text{d}x, \end{array}$$ (26)

$$\begin{array}{c} {\left[F_{a}\right]}_{i\pm 1/2}^{n}=\frac{1}{\Delta t}\int_{t_{n}}^{t_{n+1}}f_{a}\left(x_{i\pm 1/2},t\right)\text{d}t, \end{array}$$ (27)

where ${\left[Q_{a}\right]}_{i}^{n}$ is the *average charge vector* of *q* over *C*_*i*_ at time *t*_*n*_ and ${\left[F_{a}\right]}_{i\pm 1/2}^{n}$ is the *average flux vector* across the boundaries of *C*_*i*_. From now on, the square brackets notation [ ] around any numerical function is used to denote the corresponding (space or time) average related to that specific numerical function.

If ***q***(***u***(*x*, *t*)) is a smooth function, then the integral Eq (26) agrees with the value of ***q*** at the midpoint of the interval to $O(\Delta x^{2})$ [8].

The indexes outside the square bracket do not denote the spatial and time evaluation of the average vector, they are just labels that refer to the time and grid positions of the corresponding numerical values.

Substituting the definitions Eqs (26) and (27) in Eq (25) we obtain the main discretisation for the finite volume scheme usually presented in the literature (cf. [8]):

$$\begin{array}{c} {\left[Q_{a}\right]}_{i}^{n+1}={\left[Q_{a}\right]}_{i}^{n}-\frac{\Delta t}{\Delta x}({\left[F_{a}\right]}_{i+1/2}^{n}-{\left[F_{a}\right]}_{i-1/2}^{n}). \end{array}$$ (28)

Eq (28) is a numerical recipe of how to compute the mean value ${\left[Q_{a}\right]}_{i}^{n+1}$ using the average flux and charge values one time-step backwards for each grid cell *C*_*i*_. This discretisation has the same exact form as Eq (15) except for the choice of the values Eqs (26) and (27).

The advantage of this method over any finite difference scheme is that the conservative nature of the system is preserved, even across strong discontinuities such as shock waves. This is the reason as to why a finite volume scheme is often used when dealing with the physics of high energy flows where discontinuities may appear.

### Numerical flux

The flux ***f*** at Eq (27) depends on the value of *q* at every time. This is why it is impossible to integrate the average flux. Somehow, we have to find a good approximation for this integral. Moreover, the flux ***f*** inside the integral is evaluated on the boundaries *x*_*i*±1/2_ of the grid cell which, numerically speaking, has no sense because we can only approximate the values of the average charges on the midpoint of the finite volume. This set of midpoints can be “safely” considered the ones used in the finite difference mesh mentioned in the Finite differences approach section.

One way to approximate ${\left[F_{a}\right]}_{i\pm 1/2}^{n}$ is to assume that it can be obtained as a function of the cell average values of ***q*** on either side of the interface *x*_*i*±1/2_, i.e., ${\left[Q_{a}\right]}_{i\pm 1}^{n}$ and ${\left[Q_{a}\right]}_{i}^{n}$:

$$\begin{array}{c} {\left[F_{a}\right]}_{i\pm 1/2}^{n}=F_{a}({\left[Q_{a}\right]}_{i\pm 1}^{n},{\left[Q_{a}\right]}_{i}^{n}). \end{array}$$ (29)

The previous result is expected since in a hyperbolic problem the information of how ***q*** change on every cell propagates at a finite characteristic speed (see e.g. [3, 14]). The function $F_{a}$ can be thought as a *numerical flux function* for which its functional form will depend on the problem or the particular numerical scheme used to solve it.

Substitution of Eq (29) into Eq (28) yields:

$$\begin{array}{c} {\left[Q_{a}\right]}_{i}^{n+1}={\left[Q_{a}\right]}_{i}^{n}-\frac{\Delta t}{\Delta x}[F_{a}({\left[Q_{a}\right]}_{i+1}^{n},{\left[Q_{a}\right]}_{i}^{n})-F_{a}({\left[Q_{a}\right]}_{i-1}^{n},{\left[Q_{a}\right]}_{i}^{n})]. \end{array}$$ (30)

The numerical flux function is then determined by the evolution of the solution in each interface. A good first guess for the function $F_{a}$ is to relate it to the corresponding average flux function of a local (for each cell) Riemann problem [9] with two constant states on each side of the boundary.

In order to obtain an accurate numerical flux function, is important to study the behaviour of the solution based on the form and properties of the governing equation at these particular initial conditions.

### Riemann problem

Let us now consider a single conservative equation (i.e. Eq (15) with *a* = 1 only) in which the flux is written as $f\left(q\right)=\tilde{u}q$ where $\tilde{u}$ is a constant value:

$$\begin{array}{c} \frac{\partial q}{\partial t}+\tilde{u}\frac{\partial q}{\partial x}=0. \end{array}$$ (31)

This is the advection equation in which $\tilde{u}$ corresponds to the propagation velocity of *q*. Note that, since $f^{\prime }\left(q\right)=\tilde{u}$, Eq (31) is also its own quasilinear version.

The function $q\left(x,t\right)=\tilde{q}\left(x-\tilde{u}t\right)$ satisfies Eq (31) for any function $\tilde{q}$. However, it is more useful for us to describe the problem observing the behaviour of the solution *q* along *characteristic curves* in the *t* − *x* plane. To do so, we perform the time derivative of ***q***(*X*(*t*), *t*) and equate the result to zero, i.e.:

$$\begin{array}{c} \frac{\text{d}}{\text{d}t}q\left(X\left(t\right),t\right)=\frac{\partial q}{\partial t}+X^{\prime }\left(t\right)\frac{\partial q}{\partial x}=0. \end{array}$$ (32)

Direct comparison of the above equation with Eq (31), means that that the solution *q*(*X*(*t*), *t*) is constant all along the ray $X\left(t\right)=x_{0}+\tilde{u}t$, where *x*_0_ is some initial value. In the most general case, the set of all rays *X*(*t*) are called the *characteristics* of the equation.

If we consider the particular case in which the initial conditions of the problem consists on two constant states

$$\begin{array}{c} q\left(x,0\right)=\{\begin{array}{cc} q_{l}, & \text{if}\;x<0,\\ q_{r}, & \text{if}\;x>0, \end{array} \end{array}$$ (33)

where *q*_*l*_ and *q*_*r*_ are the left and right states respectively, the characteristics *X*(*t*) of (31) are then rays with slope $\tilde{u}$ in the *t* − *x* plane. With this, the solution can be written as

$$\begin{array}{c} q\left(x,t\right)=\{\begin{array}{cc} q_{l}, & \text{if}\;x-\tilde{u}t<0\;\text{or}\;x/t<\tilde{u},\\ q_{r}, & \text{if}\;x-\tilde{u}t>0\;\text{or}\;x/t>\tilde{u}. \end{array} \end{array}$$ (34)

Let us consider now a system of *m* conservative equations (i.e. *a* = 1, 2, …, *m* in Eq (15)), where *f*_*a*_ = *A*_*ab*_*q*_*b*_, i.e.:

$$\begin{array}{c} \frac{\partial q_{a}}{\partial t}+A_{ab}\frac{\partial q_{b}}{\partial x}=0, \end{array}$$ (35)

where *A*_*ab*_ is a constant *m* × *m* matrix and so, the system of conservative equations is linear. If *A*_*ab*_ is diagonalisable such that:

$$\begin{array}{c} A_{ab}=R_{ac}\Lambda_{cd}R_{db}^{-1}, \end{array}$$ (36)

where *R*_*ac*_ is the matrix of eigenvectors, with $r_{a}^{p}$ the *p*-th eigenvector, $R_{db}^{-1}$ its inverse and $\Lambda_{cd}=\text{diag}\left(\lambda^{1},\ldots ,\lambda^{m}\right)$, for λ^*p*^ the *p*-th eigenvalue. If we define the *characteristic variables* *w*_*a*_ as

$$\begin{array}{c} w_{a}\left(x,t\right)≔R_{ab}^{-1}q_{b}\left(x,t\right), \end{array}$$ (37)

it is then possible to rewrite Eq (35) as the following system of *m* advective equations:

$$\begin{array}{c} \frac{\partial w_{a}}{\partial t}+\Lambda_{ab}\frac{\partial w_{b}}{\partial x}=0. \end{array}$$ (38)

In the case of the Riemann problem, the solution for the *p*-th advective equation is $w_{p}\left(x,t\right)={\tilde{w}}_{p}\left(x-\lambda^{p}t,0\right)$, and the solution *q*_*a*_(*x*, *t*) is obtained using the definition of *w*_*a*_:

$$\begin{array}{c} q_{a}\left(x,t\right)=R_{ab}{\tilde{w}}_{b}\left(x,t\right). \end{array}$$ (39)

In this way one can think that *q*_*a*_ is a superposition of *m* waves moving with *characteristic velocities *λ^1^, λ^2^, … and λ^*m*^, respectively [14].

Another way to see this is by comparing Eq (35) with the time derivative of *q*(*X*(*t*), *t*) in (32). From this, it follows that the characteristics are curves for which their corresponding slopes are exactly the eigenvalues of the matrix *A*_*ab*_.

In order to obtain a real contribution of one of these waves to the evolution of a contiguous grid cell, the size of the control volume must be larger than the distance travelled by the wave, moving at its characteristic velocity, at a certain fixed time Δ*t*, i.e.,

$$\begin{array}{c} \lambda \frac{\Delta t}{\Delta x}<1. \end{array}$$ (40)

The quantity λΔ*t*/Δ*x* is know as the *Courant number* and the fulfilment of Eq (40) is called *Courant-Friedrich-Levy (CFL) condition*. This is a convergence requirement for several numerical methods that solve conservative equations.

The Riemann problem discussed in this subsection, is used to accurately estimate the value of the numerical fluxes at the boundaries of two contiguous grid cells as will be seen in the following section.

### Godunov scheme

Godunov in 1959 [17] proposed a numerical scheme for solving conservative equations and this method can be used in terms of the Riemann problem as follows. Consider the single Eq (31). The algorithm proposed by Godunov has the following recipe:

1. Compute the average values of the charges *q* at the time *t* = *t*_*n*_ using Eq (26) for *a* = 1 only:
  $$\begin{array}{c} {\left[Q\right]}_{i}^{n}=\frac{1}{\Delta x}\int_{C_{i}}q\left(x,t_{n}\right)\text{d}x. \end{array}$$ (41)
2. Reconstruct from ${\left[Q\right]}_{i}^{n}$ a polynomial function $\tilde{q}\left(x,t_{n}\right)$ for every value of *x*. The simplest case for this is to take a constant function:
  $$\begin{array}{c} \tilde{q}\left(x,t_{n}\right)≔{\left[Q\right]}_{i}^{n}\;\text{for}\;x\in C_{i}. \end{array}$$ (42)
  In practice [18], the value ${\left[Q\right]}_{i}^{n}$ is consider to be *q* evaluated at the midpoint of the grid cell.
3. Evolve the hyperbolic equation in an exact or approximate way by a time Δ*t* to obtain $\tilde{q}\left(x,t_{n+1}\right)$.
4. Take the average of $\tilde{q}\left(x,t_{n+1}\right)$ over *C*_*i*_ to obtain ${\left[Q\right]}_{i}^{n+1}$.
5. Go back to the first item on the list and iterate until a final time is reached.

As we discuss above, it is impossible to compute exactly the average flux ${\left[F\right]}_{i\pm 1/2}^{n}$ because we do not know the value of *q* at all times. However, if we consider a Riemann problem in the interface *x*_*i*±1/2_ between the grid cells *C*_*i*_ and *C*_*i*±1_ and apply step 3 of Godunov’s algorithm, we get that $\tilde{q}\left(x_{i\pm 1/2},t\right)$ is constant along the curves that satisfies (*x* − *x*_*i*±1/2_)/*t* = const.

In summary, if we denote by $q^{↓}\left({\left[Q\right]}_{i}^{n},{\left[Q\right]}_{i\pm 1}^{n}\right)$ the solution to the Riemann problem at *x*_*i*±1/2_, the computation of the average fluxes reduces on computing an integral over a constant function [8]. In this way, the Godunov’s algorithm can be expressed in terms of average fluxes using the following recipe:

1. Solve the Riemann problem in the interfaces *x*_*i*±1/2_ of the *C*_*i*_ grid cell in order to obtain $q^{↓}\left({\left[Q\right]}_{i}^{n},{\left[Q\right]}_{i\pm 1}^{n}\right)$.
2. Define $F\left({\left[Q\right]}_{i}^{n},{\left[Q\right]}_{i\pm 1}^{n}\right)=f\left(q^{↓}\left({\left[Q\right]}_{i}^{n},{\left[Q\right]}_{i\pm 1}^{n}\right)\right)$.
3. Apply discretisation Eq (30).

The problem with applying Godunov’s scheme on non-linear systems and considering wave propagation of characteristic waves on all interfaces, is that the characteristic velocities are not constant at all times and also they change values at different grid cells. For the case of a quasilinear system such as the one of Eq (16), an approximation has to be made. Many methods for obtaining an approximate Riemann solution have been developed and successfully implemented in classical and relativistic magnetohydrodynamic codes (see e.g. [11, 19]).

### HLL Riemann solver

**Fig 6:**
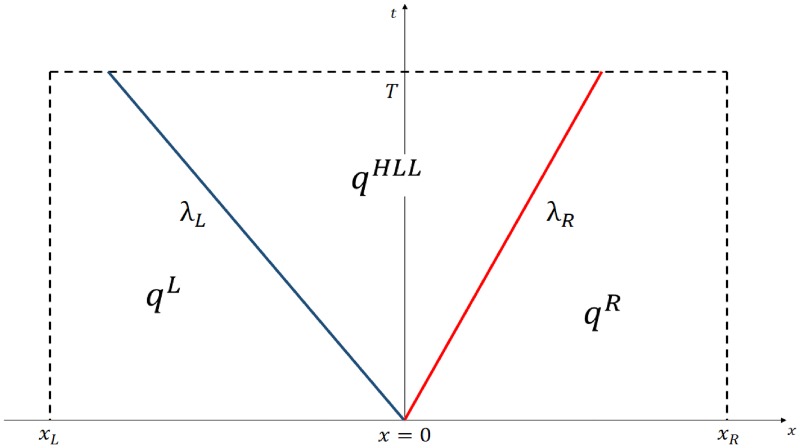
Space-time grid cell [*x*_*L*_, *x*_*R*_] × [0, *T*]. The figure shows the evolution of the 1D conservative equation solution along rays with slope λ_*L*_ and λ_*R*_, together with the intermediate state *q*^*HLL*^ generated by the HLL solver.

One of the most popular approximate Riemann solvers is the called HLL solver [20]. This Godunov’s base method considers a Riemann problem with constant states ***q***^*L*^ and ***q***^*R*^ on each side of the interface in a space-time grid cell [*x*_*L*_, *x*_*R*_] × [0, *T*] as shown on Fig 6.

Instead of following the solution of all the characteristic variables along their own characteristic velocities, the idea of the HLL approximation consists on considering the larger eigenvalues λ_*R*_ and λ_*L*_ moving across the interface to the right and left respectively. The region delimited by these characteristic rays is denoted by the state ***q***^*HLL*^.

Note that, since we are working with a system of *m* conservative equations, 2*m* characteristic rays will emerge from each interface. The values λ_*L*_ and λ_*R*_ are to be chosen taking into account all 2*m* characteristic velocities.

The approximate solution to the Riemann problem derived by this scheme has the following form (see e.g. [8] or [21]):

$$\begin{array}{c} q_{a}\left(x,t\right)=\{\begin{array}{cc} q_{a}^{L}, & \text{if}\;x/t\leq \lambda_{L},\\ q_{a}HLL & \text{if}\;\lambda_{L}<x/t<\lambda_{R},\\ q_{a}^{R}, & \text{if}\;x/t\geq \lambda_{R}, \end{array} \end{array}$$ (43)

where

$$\begin{array}{c} q_{a}^{HLL}=\frac{\lambda_{R}q_{a}^{R}-\lambda_{L}q_{a}^{L}+f_{a}^{L}-f_{a}^{R}}{\lambda_{R}-\lambda_{L}}, \end{array}$$ (44)

where ***f***^*R*,*L*^ ≔ ***f***(***q***^*R*,*L*^). One can work out the approximate solution to the flux through the interface by integrating the hyperbolic equation over the space-time domain outlined in Fig 6 and using the Rankine-Hugoniot jump condition at each characteristic ray (λ_*R*,*L*_). The final result is that [21]:

$$\begin{array}{c} f_{a}^{HLL}=\frac{\lambda_{R}f_{a}^{L}-\lambda_{L}f_{a}^{R}+\lambda_{R}\lambda_{L}\left(q_{a}^{R}-q_{a}^{L}\right)}{\lambda_{R}-\lambda_{L}}. \end{array}$$ (45)

Notice that ***f***^*HLL*^ ≠ ***f***(***q***^*HLL*^). The flux Eq (45) can be used along with the Godunov scheme to solve the local Riemann problem of to contiguous grid cells.

Let us now consider the boundary *x*_*i*−1/2_ between two control volumes *C*_*i*_ and *C*_*i*−1_ and suppose that a constant reconstruction $\tilde{q}$ from the average values of ***q*** has been made. With this, let ${\tilde{q_{a}}}^{L}\left(x_{i-1/2},t_{n}\right)≔{\left[Q_{a}\right]}_{i-1}^{n}$ and ${\tilde{q_{a}}}^{R}\left(x_{i-1/2},t_{n}\right)≔{\left[Q_{a}\right]}_{i}^{n}$ to be the reconstruction points that lay at the interface *x*_*i*−1/2_. Note that these values are going to be different if a polynomial reconstruction is made. With this, we can write the numerical flux at *x*_*i*−1/2_ used in the Godunov scheme in the following form:

$$\begin{array}{c} {\left[F_{a}^{HLL}\right]}_{i-1/2}^{n}=\{\begin{array}{ccc} f_{a}^{L}\left(x_{i-1/2},t_{n}\right), & \text{if} & 0\leq \lambda_{L},\\ f_{a}^{HLL}\left(x_{i-1/2},t_{n}\right) & \text{if} & \lambda_{L}<0<\lambda_{R},\\ f_{a}^{R}\left(x_{i-1/2},t_{n}\right), & \text{if} & 0\geq \lambda_{L}. \end{array} \end{array}$$ (46)

The flux through *x*_*i*+1/2_ is obtained in an analogous way. So, by substituting these numerical fluxes in the discretisation Eq (30), we finally get the numerical solution for the hyperbolic Eq (15) in the finite volume scheme using Godunov’s algorithm with a *high resolution* [22] approximate Riemann HLL solver:

$$\begin{array}{c} {\left[Q_{a}\right]}_{i}^{n+1}={\left[Q_{a}\right]}_{i}^{n}-\frac{\Delta t}{\Delta x}({\left[F_{a}^{HLL}\right]}_{i+1/2}^{n}-{\left[F_{a}^{HLL}\right]}_{i-1/2}^{n}). \end{array}$$ (47)

A simple way of computing ${\left[Q_{a}\right]}_{i}^{n}$ is by considering that this average value match the magnitude of ***q*** evaluated at the midpoint of the grid cell *x*_*i*_. If ***q***(*x*, *t*) is smooth, the error introduced by this approximation is of order $O(\Delta x^{2})$ [8]. In other words:

$$\begin{array}{c} q_{a}\left(x_{i},t_{n+1}\right)=q_{a}\left(x_{i},t_{n}\right)-\frac{\Delta t}{\Delta x}({\left[F_{a}^{HLL}\right]}_{i+1/2}^{n}-{\left[F_{a}^{HLL}\right]}_{i-1/2}^{n}). \end{array}$$ (48)

Many other HLL-type Riemann solvers have been developed (cf. [21]) and successfully implemented (cf. [19]) but they are beyond the scope of the present article.

### Limiters

At first approximation, the reconstruction of *q* over the grid cell was made considering a constant value ${\left[Q\right]}_{i}^{n}$ which is taken as the midpoint value of *q* of the corresponding control volume *C*_*i*_. A better way of improving the precision of the above procedure is by considering a piecewise polynomial approximation for this variable.

In the linear case, the reconstruction of *q* over *C*_*i*_ is given by

$$\begin{array}{c} \tilde{q}\left(x,t_{n}\right)=q\left(x_{i},t_{n}\right)+\sigma_{i}^{n}\left(x-x_{i}\right), \end{array}$$ (49)

where $\sigma_{i}^{n}$ is the slope of the linear reconstruction. To use the limiters together with a HLL-type Riemann solver, all we need to consider are those points of $\tilde{q}$ in each contiguous grid cells, evaluated at the interfaces *x*_*i*±1/2_. In this respect, it is not important to do a complete reconstruction of *q*. The knowledge of *q* at the boundaries is sufficient for this approximation, and so the values required to effectively evolve the solution of the hyperbolic equation over the grid cell *C*_*i*_ are:

$$\begin{array}{c} {\tilde{q}}^{L}\left(x_{i-1/2},t_{n}\right)=q\left(x_{i-1},t_{n}\right)+\frac{1}{2}\sigma_{i-1}^{n}\Delta x, \end{array}$$ (50)

$$\begin{array}{c} {\tilde{q}}^{R}\left(x_{i-1/2},t_{n}\right)=q\left(x_{i},t_{n}\right)-\frac{1}{2}\sigma_{i}^{n}\Delta x, \end{array}$$ (51)

$$\begin{array}{c} {\tilde{q}}^{L}\left(x_{i+1/2},t_{n}\right)=q\left(x_{i},t_{n}\right)+\frac{1}{2}\sigma_{i}^{n}\Delta x, \end{array}$$ (52)

$$\begin{array}{c} {\tilde{q}}^{R}\left(x_{i+1/2},t_{n}\right)=q\left(x_{i+1},t_{n}\right)-\frac{1}{2}\sigma_{i+1}^{n}\Delta x. \end{array}$$ (53)

Each pair Eqs (50 and 51) and Eqs (52 and 53), constitute a Riemann problem to be solved at the interface *x*_*i*−1/2_ and *x*_*i*+1/2_, respectively. The polynomial reconstruction are useful to accurate capture discontinuities such as shock-waves. Eqs (50)–(53) are also valid for each component of the vector ***q*** when a coupled system of conservative equations is required.

The usual way of computing *σ* is by considering some useful function based on finite derivatives of *q* over *C*_*i*_. The most used but dissipative reconstruction (also called *limiter* [8]) is the *minmod limiter* (MM) introduced in [23]:

$$\begin{array}{c} \sigma_{i}^{n}=\text{minmod}\left(m_{i-1/2},m_{i+1/2}\right), \end{array}$$ (54)

where the function *m*_*i*±1/2_ is the average slope (or the finite derivative) of *q* centred at *x*_*i*±1/2_:

$$\begin{array}{c} m_{i+1/2}=\frac{q\left(x_{i+1},t_{n}\right)-q\left(x_{i},t_{n}\right)}{x_{i+1}-x_{i}}, \end{array}$$ (55)

$$\begin{array}{c} m_{i-1/2}=\frac{q\left(x_{i},t_{n}\right)-q\left(x_{i-1},t_{n}\right)}{x_{i}-x_{i-1}}. \end{array}$$ (56)

The *minmod* function of two values *a* and *b* stands for:

$$\begin{array}{c} \text{minmod}\left(a,b\right)≔\{\begin{array}{ccc} 0, & \text{if} & ab\leq 0,\\ a, & \text{if} & |a|<|b|,\\ b, & \text{if} & |b|<|a|. \end{array} \end{array}$$ (57)

This limiter has been successfully implemented in the case of relativistic hydrodynamics (cf. [11, 18]).

The monotonic centred limiter *MC*, proposed by van Leer [24], has less dissipation than minmod near discontinuities, but has been proved to create spurious oscillations in the strong shock cases [11]. Nevertheless, it produces relatively well damped solutions that capture not too strong shock waves. The slope *σ* is written as in Eq (54) but the MC function has the following form:

$$\begin{array}{c} \text{MC}\left(a,b\right)≔\{\begin{array}{ccccc} 0, & \text{if} & ab\leq 0, & & \\ 2a, & \text{if} & \left|a|<|b\right| & \text{and} & 2|a|<|c|,\\ 2b, & \text{if} & \left|b|<|a\right| & \text{and} & 2|b|<|c|,\\ c, & \text{if} & \left|c|<2|a\right| & \text{and} & |c|<2|b|, \end{array} \end{array}$$ (58)

where *c* ≔ (*a* + *b*)/2.

**Fig 7:**
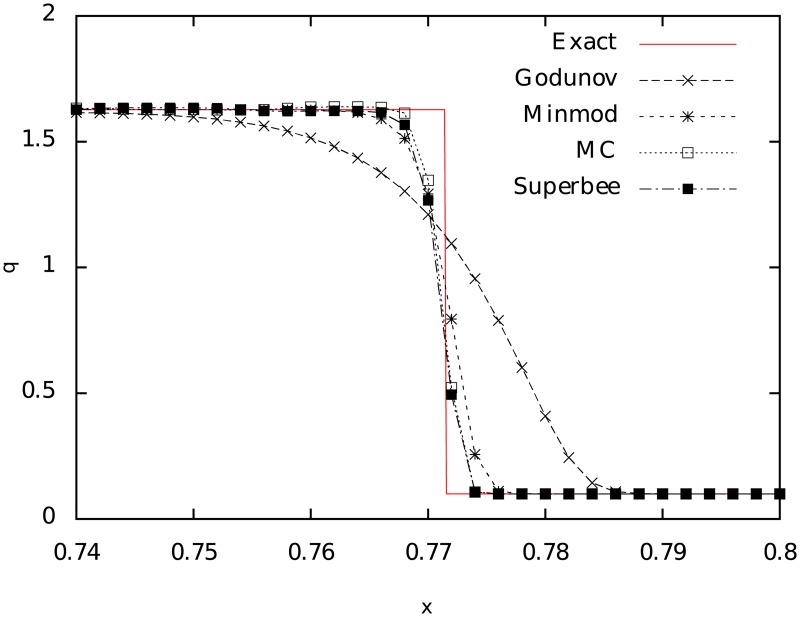
Comparison between the piecewise linear reconstructions (*minmod*, *MC* and *superbee*) with the piecewise constant one (*godunov*). As the complexity of the algorithm grows the shock capture is better, as it is shown in the figure by the superbee simulation. The graph shows the quantity *q* corresponding to the pressure as a function of the position at a fixed time *t* = 0.35 for a particular Riemann problem in a relativistic Sod shock tube that evolves from the initial value *t* = 0 in such a way that, at this time, *p* = 1.69 for *x* <0.77 and *p* = 0.1 for *x* ≥ 0.77.

Another piecewise linear reconstruction is the *superbee* limiter, also proposed by Roe in 1986 [23]. This one has a better shock-wave capture than the previous scheme as shown in Fig 7, where comparisons of the superbee limiter with the previous ones and with the piecewise constant reconstruction (*godunov*) is made. For this slope, the function is slightly more complicated than the previous ones and is given by:

$$\begin{array}{c} \sigma =\text{maxmod}(\sigma_{i}^{n(1)},\sigma_{i}^{n(2)}), \end{array}$$ (59)

where

$$\begin{array}{c} \sigma_{i}^{n(1)}=\text{minmod}(m_{i+1/2},2m_{i-1/2}), \end{array}$$ (60)

$$\begin{array}{c} \sigma_{i}^{n(2)}=\text{minmod}(2m_{i+1/2},m_{i-1/2}), \end{array}$$ (61)

and

$$\begin{array}{c} \text{maxmod}\left(a,b\right)≔\{\begin{array}{ccc} 0, & \text{if} & ab\leq 0,\\ a, & \text{if} & |b|<|a|,\\ b, & \text{if} & |a|<|b|. \end{array} \end{array}$$ (62)

Colella in 1984 [25] developed a piecewise parabolic reconstruction (PPM), that have been successfully used by many authors in both relativistic [11] and non-relativistic hydrodynamics (cf. [26]) but for the purposes of this paper, it will not be considered.

## Supporting information

S1 File Numerical vs. exact data. In the file “S1_File.tar.gz”, we attached the relevant data of the comparison between numerical simulations and exact solutions used to obtain the L_1_—norm.
(GZ) Click here for additional data file.
